# Modeling the Relationships between the Height and Spectrum of Submerged Tufa Barrage Using UAV-Derived Geometric Bathymetry and Digital Orthoimages

**DOI:** 10.3390/s21216987

**Published:** 2021-10-21

**Authors:** Jinchen He, Jiayuan Lin, Yanhao Xu

**Affiliations:** 1 Chongqing Jinfo Mountain Karst Ecosystem National Observation and Research Station, School of Geographical Sciences, Southwest University, Chongqing 400715, China; swu001204@email.swu.edu.cn (J.H.); xuyh168@email.swu.edu.cn (Y.X.); 2 Chongqing Engineering Research Center for Remote Sensing Big Data Application, School of Geographical Sciences, Southwest University, Chongqing 400715, China

**Keywords:** submerged tufa barrage, unmanned aerial vehicle (UAV), point cloud, remote sensing, spectral analysis

## Abstract

Tufa barrages play an important role in fluviatile tufa ecosystems and sedimentary records. Quantifying the height of tufa barrage is significant for understanding the evolution and development of the Holocene tufa barrage systems. However, for submerged tufa barrages, there is no low-cost non-contact method to retrieve barrage height. Generally, it is difficult to recognize small tufa barrages by means of remotely sensed satellite data, but the combination of unmanned aerial vehicles (UAV) and Structure-from-Motion (SfM) photogrammetry makes it possible. In this study, we used a fixed-wing UAV and a consumer-grade camera to acquire images of the submerged tufa barrage in Lying Dragon Lake, Jiuzhaigou National Nature Reserve, China, and estimated the height of the tufa barrage through UAV-based photogrammetric bathymetry. On this foundation, the relationship between barrage height and its spectrum was established through band ratio analysis using UAV-derived geometric bathymetry and digital orthoimages, which provided an alternative strategy to characterize the height of submerged tufa barrages. However, the spectral characteristics of submerged tufa barrages will oscillate with changes in the environmental conditions. In future research, we will consider using a dedicated aquatic multispectral camera to improve the experimentation.

## 1. Introduction

**Figure 1:**
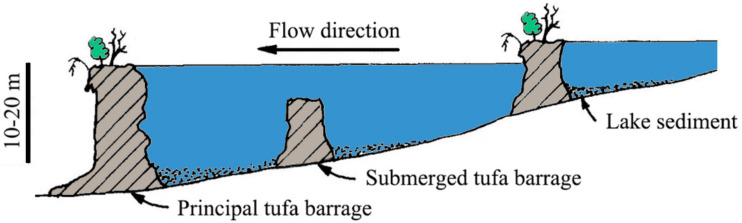
Illustration of the fluviatile barrage model and submerged tufa barrage (Adapted from Ref. [3]. 1990, Pedley).

Tufa refers to the secondary carbonate limestone formed by the deposition of karst springs, rivers and lakes at the surface or in caves under the conditions of ambient temperature [1,2]. Sedimentary models of tufa can be divided into the spring tufa model, the paludal tufa model, the lacustrine tufa model and the fluvial tufa model [3,4]. The fluviatile barrage model is the main form of tufa deposition in fluviatile tufa systems, accompanied by the appearance of tufa barrages and tufa lakes. These barrages affect the convergence and distribution of water flow in the tufa lake group, which determines the ecological landscapes of the tufa system, so tufa barrages play an important role in fluviatile tufa ecosystems. Pentecost [5] described fluvial tufa barrages as being perpendicular to the direction of local flow, with somewhat sinuous crests. Because tufa barrages are generally below the water surface, they are often called reefs in freshwater systems [1]. However, due to the differences in the tufa deposition rate [6], the growth in height of the principal tufa barrage downstream can leads to a rise in upstream lake water levels, thus submerging the secondary tufa barrage upstream (as shown in Figure 1). As a result, this poses a heavy challenge for monitoring and quantifying the height of the submerged tufa barrage, which is indispensable to understanding the evolution and development of the Holocene tufa barrage systems.

Conventional underwater environmental surveys are mainly based on contact measurement methods such as sonar, usually including single/multi-beam echosounder systems (SBES/MBES) [7]. Generally, remote sensing refers to any non-contact technique by which the object’s space can be observed [8]. Due to its advantages of low cost and high coverage [9,10], remote sensing satellite technology has been widely used in geomorphological, hydrological and ecological research, including fluviatile tufa systems. Florsheim et al. [11] utilized QuickBird images and SRTM digital elevation models (DEM) to carry out the detection of large-scale travertine dams, which showed the unique advantages of remote sensing satellite technology in research into travertine dams. However, the relatively low spatial resolution makes it difficult to identify small tufa barrages from satellite imagery, not to mention topographic data such as the barrage height. On the other hand, airborne lidar (light detection and ranging) has the characteristics of high precision and high spatial resolution. Profe et al. [12] testified to the applicability of airborne lidar as a new remote sensing technology for characterizing tufa barrages in relation to channel bed morphology in a small karstic river. However, considering the cost and load, airborne lidar is not suitable for the measurement of tufa barrage morphology with high frequency. In addition, the near-infrared energy utilized by lidar instruments is easily absorbed by the water [13], thus the submerged tufa barrage cannot be detected and measured, except by bathymetric lidar. On the other hand, for optical remote satellite sensing, the submerged topography can be converted from satellite-derived bathymetry. Therefore, bathymetric measurements are significant for the estimation of the height of a submerged barrage, and the elevation information of the tufa barrage is indirectly reflected through the water depth.

In recent years, with the popularization of unmanned aerial vehicle or system (UAV, UAS, or “drone”) technology [14,15,16] and the development of Structure-from-Motion (SfM) photogrammetry [17,18], researchers have carried out multiple studies using the images acquired by the consumer-grade cameras carried on UAVs, such as studies on bank erosion [19], coastal monitoring [20,21], biomass estimation [22] and aquatic plant modeling [23]. Over the last decade, low-cost UAVs or drones have proven to be a new and effective tool that can collect high-quality elevation data in inaccessible areas, giving geoscientists a relatively high-resolution insight into geomorphic environments [16]. However, only a small number of researchers have exploited UAV-acquired RGB (red–green–blue) images to undertake quantitative research on submerged topography and bathymetric surveys [13,24,25]. Considering the light refraction occurring at the air–water interface, the actual water depth can be achieved through proper refraction correction of the initial water depth obtained from UAV-based SfM photogrammetry. Woodget et al. [25] conducted a simple refraction correction and quantitative evaluation of the shallow fluvial topography in a submerged area. Their study showed that UAV remote sensing is capable of estimating the water depth and underwater topography of shallow and clear waters under sufficient illumination conditions. In addition, the combination of UAV optical imagery and bathymetric inversion algorithms enables the water depth value to be estimated by means of RGB sensors [26] or multispectral cameras [27] embedded in a UAV.

Through summarizing and comparing the above bathymetric technical methods (see Table 1), it can be seen that UAV-based remote sensing technology is one of the most powerful tools for studying a small tufa system. First, in terms of spatial resolution, the UAV platform can capture clearer images compared with remote sensing satellite technology. Second, UAVs will not touch the water body, unlike sonar technology, so they will hardly interfere with the aquatic environment. Third, compared with airborne lidar bathymetry, the consumer-grade camera carried by an UAV is cheaper. Additionally, the operability and data processing workflow of UAVs are easier to be adopted by users. Although both UAV-derived spectral and geometric bathymetry can provide low-cost and high-resolution bathymetric data, the former needs the measured values as training data, while the latter needs complex three-dimensional (3D) reconstruction. Therefore, these two methods have their own unique advantages and disadvantages, and have a certain complementarity [28].

However, no research has been reported on the application of UAV-acquired optical imagery for estimation of the height of submerged tufa barrage so far. This will be the first attempt to quantify the height of a submerged tufa barrage using UAV-derived geometric bathymetry, and to analyze the relationship between its height and spectrum. In general, our work addresses a topic of increasing importance through an original spatial and spectral strategy for 3D modeling of submerged tufa barrages without coming into contact with the study object. In this study, we used a fixed-wing UAV system equipped with a consumer-grade camera to acquire RGB images of the submerged tufa barrage in Jiuzhaigou National Nature Reserve in China. Based on the UAV-acquired RGB imagery and SfM photogrammetry, we estimated the height of the submerged tufa barrage by proper refraction correction, and analyzed the relationship between its height and spectrum. Finally, we further explained the impact of environmental factors on the optical properties of submerged tufa barrages.

## 2. Study Area

**Figure 2:**
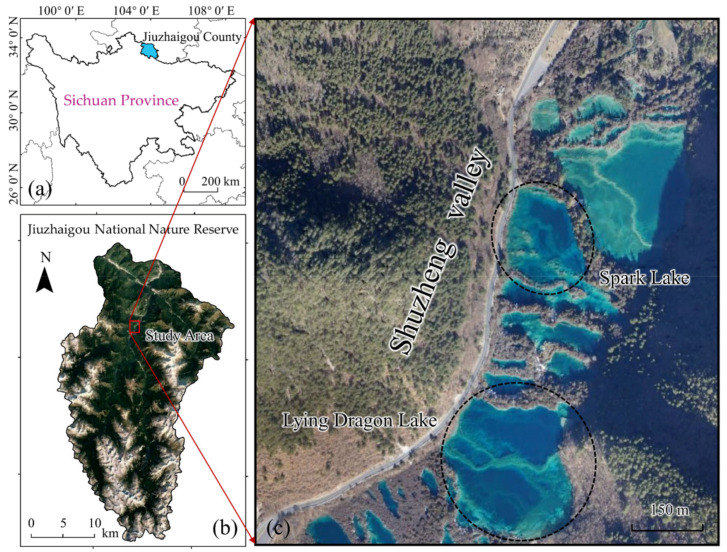
Location of the study area. (**a**) Jiuzhai County in Sichuan Province, (**b**) Jiuzhaigou National Nature Reserve, and (**c**) the main tufa lakes in Shuzheng Valley.

Our study was conducted in Jiuzhaigou National Nature Reserve of Jiuzhaigou County, Sichuan Province, southwestern China (Figure 2), which is famous for its natural karst landscape in Jiuzhai Valley. The valley is composed of Rize Valley and Zechawa Valley upstream, and Shuzheng Valley downstream. Water from upstream flows from south to north through Shuzheng Valley, and finally into the Baihe River, a tributary of the Yangtze River [11].

There is a huge fluviatile tufa system in Jiuzhai Valley, and tufa lakes, tufa beaches and tufa waterfalls are scattered in this system. The tufa landscape in the valley has been a karst accumulation landform since the Holocene, which was formed on the basis of various moraine landforms formed by the shrinkage and retreat of ancient glaciers [29]. As the years proceeded, tufa layers piled up to form different-sized tufa barrages on the riverbed, and then developed into tufa lakes and waterfalls. Beyond doubt, fluviatile tufa barrages perpendicular to the direction of local flow are prominent features of Jiuzhai Valley.

Our study object is located in Lying Dragon Lake in Shuzheng Valley, which is adjacent to Spark Lake to the north (Figure 2c). The lake is 2215 m above sea level, with an average water depth of 22 m and an area of 61,000 m^2^. An opalescent submerged tufa barrage shaped like a Chinese dragon lies across the lakebed, which is the origin of the lake’s name. The barrage is also formed by the deposition of tufa sediments mainly made of calcium carbonate substance on the lakebed. When we look down from the sky, the submerged tufa barrage has an east–west trend and is divided into two branches on the west side. However, this quantitative description of the barrage morphology is insufficient, especially regarding its height. For the purpose of protecting the natural landscape, the scenic area management department prohibits contact between humans and the water. Therefore, we estimated the height of the submerged tufa barrage for research using UAV-based SfM photogrammetry.

## 3. Methods

**Figure 3:**
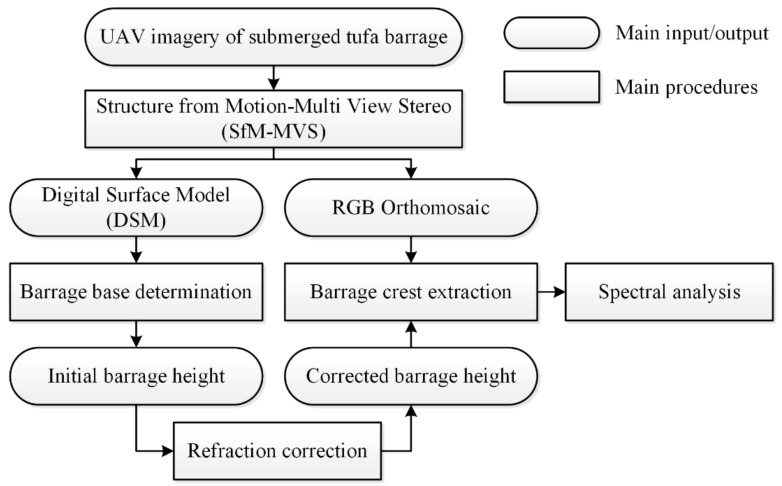
Flowchart for processing UAV images of the submerged tufa barrage.

In this part, we will introduce our methods in four modules, including UAV data acquisition, UAV data processing, refraction correction of tufa barrage height, and through-water spectral analysis. First of all, UAV data acquisition is the basis of the whole research, while UAV data processing is the key to extracting the terrain information of the submerged tufa barrage. Through the processing of UAV-acquired RGB images, the initial digital surface model (DSM) and an orthomosaic can be created [30]. On this basis, certain post-processing steps for the initial output are necessary, including refraction correction for the initial tufa barrage height and pixel gray-level extraction of the dam crest. Finally, by establishing the relationships between the height and spectrum of the submerged tufa barrage, we can indirectly reflect the change in the height of the tufa barrage through the spectral information of UAV-acquired optical images. The whole workflow of our study is shown in Figure 3.

### 3.1. UAV Data Acquisition

**Figure 4:**
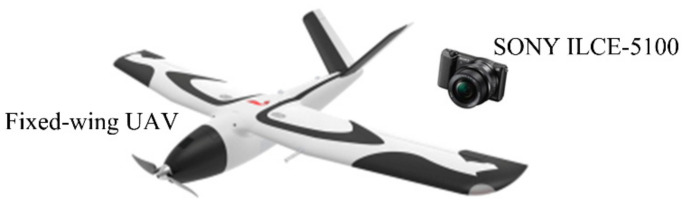
UAV platform and sensor for image acquisition.

In the process of data acquisition, we used a fixed-wing UAV equipped with a consumer-grade digital camera to carry out the flight (Figure 4). The UAV platform had a wing width of 1.2 m, a fuselage length of 0.8 m and a working weight of 4.2 kg. The two motor models used by the UAV were SunnySky V3508 units with a KV value of 580. The digital camera was a SONY ILCE-5100 with a resolution of 6000 × 4000 pixels, a focal length of 20 mm, a size of 75 mm × 63 mm × 50 mm, and a weight of 192 g, including the lens.

The UAV images of the submerged tufa barrage were collected in good light on the morning of 9 December 2016. The altitude of the flight was 500 m relative to the take-off point. The course overlap of the acquired images was 80% and the side overlap was 70%. Each position of our study area was covered by at least five photos. The spatial resolution of the images was 10 cm, which was much higher than that of general satellite images. During the flight mission, the reflected light intensity and texture information of the whole submerged tufa barrage was captured by the camera and clearly recorded in the optical images.

### 3.2. UAV Data Processing

**Figure 5:**
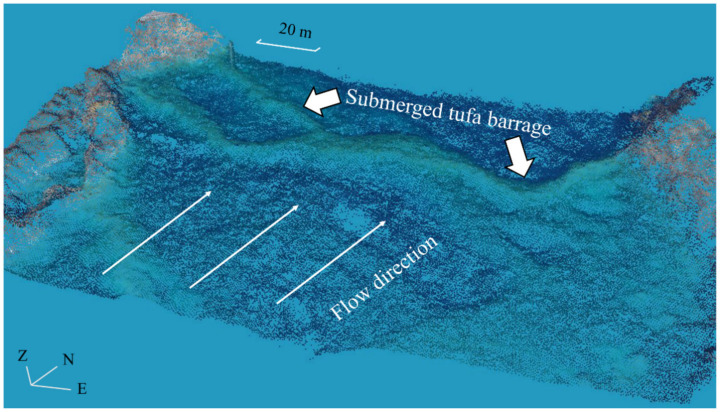
Dense point cloud after the SfM-MVS workflow; the submerged tufa barrage is marked.

In this study, we imported the photos taken by the UAV-borne digital camera into Pix4Dmapper software, and applied its built-in Structure-from-Motion algorithm and Multi-view Stereo algorithm, namely SfM-MVS. The processing flow of SfM-MVS amis to automatically extract the key points in the successive overlapping images, and then all the images are matched where the key points identified in the images appear. Given enough images and matching key points, the SfM algorithm performs bundle adjustment to minimize the reprojection error, calculates camera poses and parameters, and generates a sparse point cloud. The camera’s inner parameters (including focal length, principal point coordinates, radial distortion parameters, tangential distortion parameters, etc.) can be optimized in the bundle adjustment [31]. Next, using the camera poses and parameters obtained from the SfM workflow, the MVS algorithm is used to generate a dense point cloud. The dense point cloud with spectral information from the input images represents the main output of the SfM-MVS workflow (as shown in Figure 5). The subsequent processing steps usually include the production of DSM and RGB orthomosaics [32], which are finally presented in Tagged Image File Format (TIFF) form.

**Figure 6:**
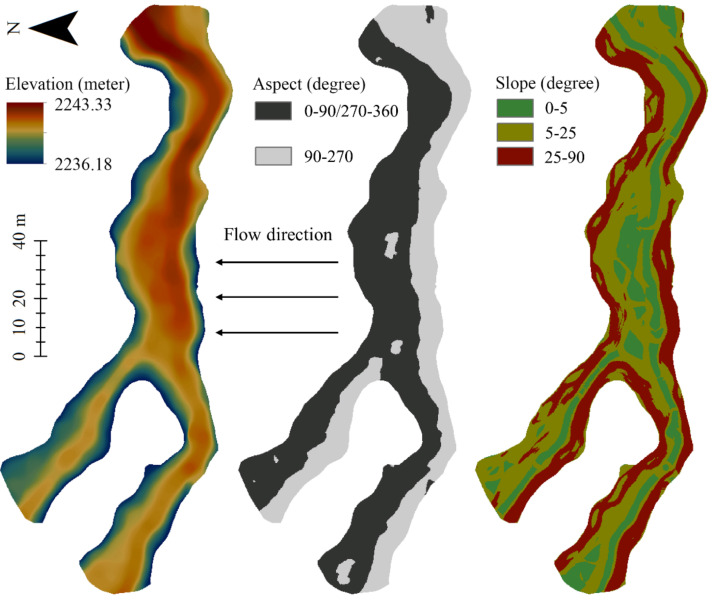
Elevation, aspect and slope of the submerged tufa barrage.

In the generated DSM, we extracted the extent of the submerged tufa barrage, which was the initial topography of the tufa barrage, namely the initial DEM. Based on the initial DEM, the corresponding aspect and slope of the barrage were further computed and generated (Figure 6). As mentioned earlier, fluvial tufa barrages are often perpendicular to the direction of water flow [5]. In our case, the water flows from south to north, so the barrage body is determined to have an east–west trend. On the other hand, because the tufa barrage in our study is located in the Northern Hemisphere, the slope with an aspect angle between 90° and 270° is a sunny slope; otherwise, it is shady slope. The boundary between the sunny slope and the shady slope represents the highest the point of tufa barrage crest. For determination of the tufa barrage height, the lowest elevation of the lakebed of Lying Dragon Lake (2215 m) was used as the barrage base [33]. The height of the barrage body is the difference in elevation between the highest local point of the barrage crest and the barrage base, which is the initial height of submerged tufa barrage. Finally, the initial height was extracted from the initial DEM of the tufa barrage.

### 3.3. Refraction Correction

However, due to the refraction of light occurring at the interface of air and water, the initial height of the submerged tufa barrage often deviates, and thus appropriate refraction correction is needed. In order to convert the height value into water depth value, we collected the elevation of the lake water boundary’s altimetric point. As a result, the value of 2245 m was preliminarily determined as the elevation of the lake surface. On this basis, the distance from the water surface to the barrage crest was calculated, which was the initial water depth. As shown in Equation (1), using a simple refraction correction model [34], a constant correction coefficient is given for the initial water depth, which is generally the refractive index 1.34 of pure water. Woodget et al. [25] proved that this method can greatly reduce the deviation bathymetric measurements and does not need more input parameters. Finally, the corrected water depth was subtracted from the relative elevation of the water surface to obtain the corrected height of the submerged tufa barrage.

*h* = *n* × *h*_0_ (1)

where *n* is the refraction index of water, *h*_0_ is the initial water depth before refraction correction and *h* represents the corrected water depth.

In previous work, we evaluated the water depth measurements of the neighboring tufa lake, namely Spark Lake (as shown in Figure 2c), after using the same refraction correction method [35]. It was found that the corrected water depth was in good agreement with the reference value within the range of 12 m. Considering that the UAV images used in the two studies were obtained at the same time and on the same equipment, the illumination conditions and sources of systematic error of the two studies are basically consistent. Meanwhile, in this research, the water depth at the crest of submerged tufa barrage was completely within 12 m, which is not more than the maximum bathymetric value evaluated by previous work. To sum it up, the corrected water depth or height of the submerged tufa barrage has a certain credibility.

### 3.4. Through-Water Spectral Analysis

Generally, in addition to the geometric analysis method used to obtain the tufa barrage height, the spectral analysis method can also be used to monitor the change in height of a submerged tufa barrage [36]. The latter has the higher usability and portability of a remote sensing platform; meanwhile, the height value of a submerged tufa barrage can be directly estimated from imagery without complex SfM-MVS processing. Through establishing the relationships between the height and spectrum of a submerged tufa barrage, the barrage morphology can be characterized in real time. However, it is quite difficult to measure continuous and high-resolution height reference data of tufa barrages in the field. In our study, taking the corrected water depth or height of the submerged tufa barrage as the reference value [28], we extracted the digital number (DN) values (red band, green band and blue band) of the barrage crest pixels to carry out a through-water spectral analysis. Because the crest of the tufa barrage is within the range of the Secchi depth of the tufa lake, this method of optical remote sensing is feasible [37]. In this study, we used the optimal band ratio analysis (OBRA) as a standard method to carry out through-water spectral analysis [38]. The simplified expression of the model is shown in the following equation:

*H* = *a* × *ln*(*Band*_1_/*Band*_2_) + *b* (2)

where *H* represents the height of the submerged tufa barrage; *Band*_1_ and *Band*_2_ are the digital number values of the red, green and blue bands; and *a*, *b* are empirical coefficients.

By means of training different regression models using Equation (2), this method determines the optimal band combination of the ratio model and provides the strongest coefficient of determination (R^2^) to infer the tufa barrage height. In our study, in order to show the relationship between the ratio value of different band combinations and the submerged barrage height, we used the correlation coefficient (R) as the measurement index to evaluate the regression analysis of ln(green/red), ln(blue/red) and ln(blue/green). The final purpose was to determine the optimal band combination which could best reflect the change in height of the barrage crest, so as to accurately estimate the height of the submerged tufa barrage.

## 4. Results

### 4.1. Tufa Barrage Morphology

As shown in Figure 6, the barrage body presents a nested distribution with a branch structure on its west side, so it looks like a Y-shape as a whole. Besides, the barrage is perpendicular to the flow direction and has a curved shape. In the direction of water flow, the width of the barrage is thinner in the concave parts and thicker in the convex parts. After rough measurement, it was found that the total length of the main barrage is about 240 m, and the widest place can reach 20 m. In terms of aspect and slope, the main barrage is obviously divided into a sunny slope (aspect angle is between 90° and 270°) and a shady slope, and the barrage crest was relatively flat (the slope angle is less than 5°), especially in the middle arc-shaped area.

### 4.2. Estimated Barrage Height

**Figure 7:**
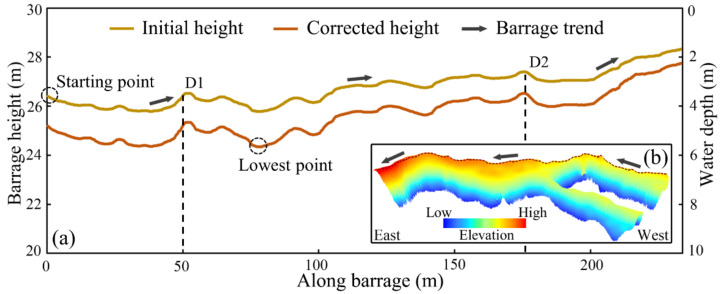
Refraction correction of the submerged barrage height. (**a**) Barrage crest height before and after correction, and (**b**) three-dimensional (3D) effect of the tufa barrage. The dotted line represents the location of the barrage crest.

In our study, the height and position of the tufa barrage crest is shown in Figure 7a,b. As shown in the main figure, the corrected barrage height generally decreased compared with the initial height, and the deeper the water, the more the barrage height decreased. The lowest height of the barrage decreased from 26 m to nearly 24 m. Additionally, the fluctuation range of the tufa barrage height expanded from almost 2 m to 4 m (see Figure 7a). In the attached figure, the 3D effect of the tufa barrage is shown, in which the dotted line represents the location of the barrage crest (Figure 7b). On the whole, the trend of the tufa barrage increases gradually from west to east; in other words, the submerged tufa barrage on the right side of the flow direction is higher than that on the left side.

However, because both sides of the barrage are connected with the banks, the elevation of the tufa barrage is easily affected by external factors, such as landslides and collapses on both sides of the streamway. Therefore, our focus was mainly on the middle part of the barrage. Along the barrage from the west side, there are two relative peaks about 50 m (D1) and 175 m (D2) away from the starting point. The height variation between the two peaks is considered to be mainly affected by tufa deposition and less by external factors. As a result, the spatial variation of the tufa barrage height is still increasing gradually from west to east within this section.

### 4.3. Band Ratio Analysis

**Figure 8:**
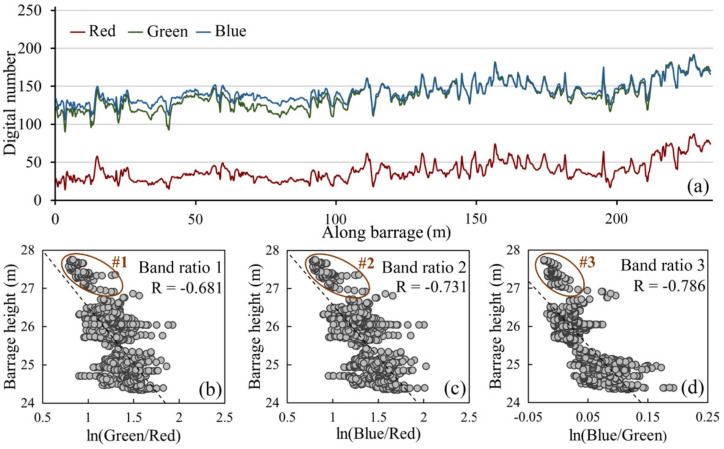
Through-water spectral analysis: (**a**) digital number values of the red, green and blue bands, (**b**) ln(green/red), (**c**) ln(blue/red) and (**d**) ln(blue/green). The ellipses delineate abnormal scatter points.

Along the crest of the submerged tufa barrage from west to east, the DN values of the red, green and blue bands are shown in Figure 8a. In contrast to Figure 7a, it is not difficult to see that the DN values increased with the increase in tufa barrage height as a whole. In general, compared with the blue and green bands, the red band had a relatively lower DN value, which was about 100 lower on average. On the other hand, the DN value curves of the blue band and the green band showed great similarity, and they even coincided at the local peak. Nevertheless, the gray-level variation trends of the red, green and blue bands were basically the same.

Based on band calculation and regression analysis, the scatter plots of the multiple band combinations, including ln(green/red), ln(blue/red) and ln(blue/green), with the corrected barrage height are shown in Figure 8b–d. Among all the models, Band ratio 1 (Figure 8b), Band ratio 2 (Figure 8c) and Band ratio 3 (Figure 8d) all showed obvious negative correlations. Among these, the correlation coefficient of Band ratio 3 was the largest (0.786), followed by Band ratio 2 (0.731), while Band ratio 1 was the smallest (0.681). Although the overall differences in the above models are not large, there is a prominent difference in the relatively high-value area of barrage height. The deviation of the blue-green band ratio model (#3) is significantly greater than that of the green–red (#1) and blue-red band combinations (#2). It can be seen that the contribution of the red band in shallow water area is higher than those of the blue and green bands. However, the overall correlation of the ratio model of the blue and green bands is stronger, especially in the relatively low-value area of barrage height. It also means that the blue and green bands have better penetration into the lake water with the increase in water depth.

## 5. Discussion

### 5.1. Factors Affecting Submerged Tufa Barrage Deposition

In the areas where tufa deposition is particularly active, the height of the tufa barrage can reach 45 m [39]. Meanwhile, the corrected height of the submerged tufa barrage is about 25 m in our case. Generally speaking, temperature, slope, calcium concentration, biological synergy and hydrodynamic conditions are the key factors affecting tufa deposition [11,40,41]. However, under the condition of low calcium concentrations in Jiuzhaigou, water quality, hydrodynamic conditions and biological synergy are undoubtedly the main factors of tufa deposition [11,42]. Additionally, abundant precipitation and the melting water of alpine ice and snow also provide a suitable aquatic environment and hydrodynamic conditions for tufa deposition in the valley. Thus, it is possible to generate large-scale tufa barrages, including submerged secondary ones.

In our case, in addition to the general height of the submerged tufa barrage, the spatial distribution characteristics of the sediment (i.e., the fluctuation of the crest height) of the tufa barrage were also the focus of our attention. Because there is no great difference in temperature and slope of the lake where the barrage is located, and the influence of underwater biological action is inconspicuous, the flow is the critical factor influencing the deposition of the tufa barrage. Due to the effects of the deflection force of the Earth’s rotation, the suspended tufa matter carried in the water tends to concentrate towards the right bank in the Northern Hemisphere. Moreover, the flow of lake water is extremely slow; hence, its erosion on the right side is quite limited. The difference between the deposition and erosion resulted in the right side of the tufa barrage being obviously higher than the left side in the direction of water flow.

Conversely, the growth or reduction of tufa barrages will also act on environmental, geological, hydrological and other processes. Firstly, the most important control on karst facies development is the primary positioning of the tufa barrages [43]. Secondly, tufa barrages can slow down the flow velocity of water and even block the flow [11]. Finally, if the submerged tufa barrage is exposed, vegetation will grow above the barrage, which will affect the ecological landscape of the fluviatile tufa system. Furthermore, tufa deposits are extremely valuable materials for recording paleohydrology and the paleoclimate, which is of great significance for the reconstruction of the paleoenvironment [44]. Therefore, tufa barrages play an important role in the whole fluviatile tufa ecosystem and the sedimentary records.

### 5.2. The Relationship between Barrage Height and Spectrum

**Figure 9:**
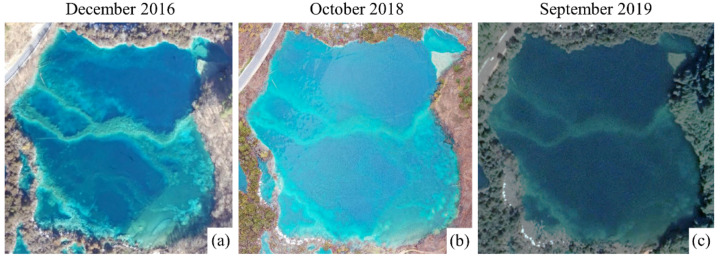
Orthomosaics of Lying Dragon Lake from a fixed-wing (**a**) and a rotary-wing UAV (**b**), and Google Earth (**c**). The shooting date of the images is marked at the top.

Generally speaking, the intensity of the optical signal will attenuate rapidly with the increase in water depth due to absorption and scattering [45,46]. Based on the radiative transfer process of light through water, the bathymetric information of rivers with clear water can be estimated from optical images [38]. In our case, the research object was the submerged tufa barrage in the lake, and its height was converted from the water depth. Consequently, it was necessary that the water surface elevation of the lake should remain relatively stable. As shown in Figure 9a–c, there was little change on the water surface of Lying Dragon Lake observed in different years and months. Thus, the height information of the submerged tufa barrage can be estimated from geometric bathymetry using UAV-based optical remote sensing.

According to the spectral response characteristics of the lake water to different spectral bands, the absorption of the optical signal by the water body is actually selective [46]. In the visible bands, the red band is largely absorbed in shallow water, followed by the green and blue bands. Thus, as the depth increases, radiance decreases in three spectral bands but more rapidly in the red band, with stronger attenuation. Although the longer wavelength (red band) was more sensitive to changes in depth, strong absorption in the spectral band limits the range of depths over which this band would be useful [47]. Therefore, for the barrage body close to the water surface, the combination of the red band and green band is more suitable for estimating the height of a submerged tufa barrage. On the contrary, if the tufa barrage is located in a deep-water area, it is more reasonable to choose the combination of the blue band and green band, theoretically. Furthermore, the band ratio model of different band combinations can not only accurately estimate the height of the barrage but also avoid the influence of different bottom types on the height estimation [36,38,48]. As a result, the selection and combination of the three visible bands are significant for estimating the height of submerged tufa barrages.

Nevertheless, the spectral characteristics of a tufa barrage will also oscillate with the change in seasons, solar elevation angle and meteorological conditions. Even on remote platforms at different heights, the spectral information captured by remote sensors will be different (see Figure 9). On the other hand, the shadow formed by mountains or trees shelter will also affect the spectral characteristics of the barrage. In general, the greater the solar elevation angle, the more radiation passing through the atmosphere into the tufa system, and the more energy the sensor will capture. UAV-based passive optical remote sensing of tufa lakes mainly involves the measurement of visible bands of the reflected solar energy that has interacted with the water column and the lakebed [38], sometimes affected by specular solar reflections (i.e., sun glint) or caused by water surface turbulence [28,47]. As a result, the change in environmental factors is often uncertain, which leads to alteration of the spectral characteristics of a tufa barrage. In order to limit the change in solar energy, data acquisition should take place at the same solar time during the same seasonal period [49]. On the other hand, a large number of optically significant components such as suspended matter, chlorophyll, and colored dissolved organic matter combine to determine the inherent optical properties of the submerged tufa barrage [47]. For instance, selective absorption of the blue band by planktonic algae will change the lake color into green or red [50]. Therefore, the relationship between the height and spectrum of the submerged tufa barrage is actually a dynamic process.

## 6. Conclusions

Tufa deposits are extremely valuable materials for the reconstruction of the paleoenvironment, paleohydrology and paleoclimate. In this study, we used a fixed-wing UAV and a consumer-grade camera to produce geometric bathymetry and digital orthoimages of Lying Dragon Lake, which is located in Jiuzhaigou National Nature Reserve in China. We then estimated the height of the submerged tufa barrage in the lake and analyzed the relationship between its height and spectrum based on band ratio models. As a result, the barrage height and its spectral characteristics were analyzed and evaluated accordingly.

Through the research and analysis, we found that the environmental conditions in Jiuzhai Valley are extremely suitable for the growth of tufa barrages. The height of the submerged tufa barrage in Lying Dragon Lake is more than 20 m. In terms of spatial distribution, the height of the barrage on the right side is significantly higher than that on the left side due to the effect of water flow. According to the spectral analysis, there is a significant correlation between the tufa barrage height and band ratio values. The combination of the blue band and green band had the best overall performance and is more suitable for deep water, while the red band performed better in shallow water. Consequently, the selection and combination of the visible wavebands are significant for estimating the height of submerged tufa barrages.

Admittedly, our research provides the possibility of estimating the height of a submerged tufa barrage directly from the UAV-derived digital orthoimage without complex 3D reconstruction. However, the spectral characteristics of submerged tufa barrages will oscillate with changes in environmental conditions. Therefore, further research should take the reliability and robustness of the relationship between barrage height and the spectrum under different environmental conditions into account, and improve our experimentation with other multispectral cameras.

## Figures and Tables

**Table 1:** Comparison of conventional bathymetric methods (single/multi-beam echosounder systems, SBES/MBES), satellite-derived bathymetry, airborne lidar bathymetry, UAV-based Structure-from-Motion (SfM) photogrammetry and UAV-RGB band-derived bathymetry. Both satellite and UAV-derived spectral bathymetry need measured sample data to train the models, so they have a certain level of interference in the aquatic environment.

	Spatial Resolution	Interference Intensity	Operational Complexity	Costs
SBES/MBES	medium	high	high	high
satellite airborne lidar UAV-SfM UAV-RGB bands	low high very high very high	medium low low medium	low very high medium low	very low very high low low

## Data Availability

Not applicable.
